# Comparison of TLD calibration methods for ${\;}^{192}\text{Ir}$ dosimetry

**DOI:** 10.1120/jacmp.v14i1.4037

**Published:** 2013-01-07

**Authors:** Annette Haworth, Duncan J. Butler, Lisa Wilfert, Martin A. Ebert, Stephen P. Todd, Anna J.M. Hayton, Tomas Kron

**Affiliations:** ^1^ Dept. Physical Sciences Peter MacCallum Cancer Centre Melbourne; ^2^ School of Applied Sciences RMIT University Melbourne; ^3^ Australian Radiation Protection and Nuclear Safety Agency Yallambie; ^4^ Calvary Mater Newcastle Hospital Newcastle; ^5^ School of Mathematical & Physical Sciences University of Newcastle Newcastle; ^6^ Dept. Radiation Oncology Sir Charles Gairdner Hospital Perth; ^7^ School of Physics University of Western Australia Crawley Australia

**Keywords:** brachytherapy, thermoluminescence dosimetry, LiF:Mg, Ti, calibration

## Abstract

For the purpose of dose measurement using a high‐dose rate ${\;}^{192}\text{Ir}$ source, four methods of thermoluminescent dosimeter (TLD) calibration were investigated. Three of the four calibration methods used the ${\;}^{192}\text{Ir}$ source. Dwell times were calculated to deliver 1 Gy to the TLDs irradiated either in air or water. Dwell time calculations were confirmed by direct measurement using an ionization chamber. The fourth method of calibration used 6 MV photons from a medical linear accelerator, and an energy correction factor was applied to account for the difference in sensitivity of the TLDs in ${\;}^{192}\text{Ir}$ and 6 M V. The results of the four TLD calibration methods are presented in terms of the results of a brachytherapy audit where seven Australian centers irradiated three sets of TLDs in a water phantom. The results were in agreement within estimated uncertainties when the TLDs were calibrated with the ${\;}^{192}\text{Ir}$ source. Calibrating TLDs in a phantom similar to that used for the audit proved to be the most practical method and provided the greatest confidence in measured dose. When calibrated using 6 MV photons, the TLD results were consistently higher than the ${\;}^{192}\text{Ir}-\text{calibrated}$ TLDs, suggesting this method does not fully correct for the response of the TLDs when irradiated in the audit phantom.

PACS number: 87

## I. INTRODUCTION

Thermoluminescent dosimeters (TLDs) were recently used in an audit of HDR brachytherapy centers in Australia. To determine the absolute dose delivered to the TLDs, it was first necessary to calibrate the TLDs to establish the relationship between TLD reading and absorbed dose. A literature search of high dose rate brachytherapy audits and brachytherapy *in vivo* projects found that most studies calibrated the TLDs using a ${\;}^{60}\text{Co}$ or megavoltage source and an energy correction factor was applied to account for the difference in response of the TLDs in the ${\;}^{192}\text{Ir}$ field.^(^ ^1^ ^–^ ^2^ ^)^ The energy correction factor is dependent on the reference energy and the characteristics of the TLD including composition and size, and the TLD annealing and read‐out protocols used.^(^ ^3^ ^–^ ^4^ ^)^ There is much debate over the contribution of this energy response, with values ranging from no energy response to more than 4.5% for the commonly used TLD‐100 when the response to ${\;}^{192}\text{Ir}$ is compared with ${\;}^{60}\text{Co}$.^(^ ^2^ ^–^ ^3^ ^,^ ^5^ ^–^ ^6^ ^)^ Furthermore, there is some debate on the variation of this energy response factor with distance from the source, with Meigooni et al.^(^ ^2^ ^)^ suggesting this factor may range in value by up to 8.5% over a distance of 1–100 mm in water from the source due to the shift of the photon spectrum to lower energies with increasing depth. In contrast, Karaiskos et al.^(^ ^6^ ^)^ determined this variation with distance (up to 150 mm) to be less than 3%.

TLDs to be used for ${\;}^{192}\text{Ir}$ dosimetry are commonly calibrated with a ${\;}^{60}\text{Co}$ or a megavoltage source because these sources can be readily accessed, can precisely deliver a prescribed dose of radiation using well‐established dosimetry (through dosimetry formalisms such as TRS‐398^(^ ^7^ ^)^ and AAPM‐51^(^ ^8^ ^)^, and it is possible to produce a uniform radiation field on a flat surface enabling several TLDs to be irradiated to the same dose in a single irradiation. In contrast, using a ${\;}^{192}\text{Ir}$ source from one of the commonly used high‐dose rate afterloaders to calibrate the TLDs requires careful consideration of geometry due to the high dose gradients surrounding the source. Whilst the dose at a known distance and angle from the source axis can be calculated using the TG‐43 formalism^(^ ^9^ ^)^ and published data for the reference source, confirming the dose calculation with measurement using an ionization chamber is not simple. No standard formalism exists for measurement of absorbed dose from a ${\;}^{192}\text{Ir}$ source with ionization chambers in common use in radiotherapy. Absorbed dose‐to‐water primary standards for ${\;}^{192}\text{Ir}$ are still under development. The calculation and/or measurement of absorbed dose is made more complicated if non‐water equivalent materials are used within the irradiated field.

The purpose of this project was to investigate a range of methods for calibrating TLDs for use with a high‐dose rate ${\;}^{192}\text{Ir}$ source. Whilst our project was concerned with identifying the most suitable method for the Australian Brachytherapy Audit, the methodology could be adapted to other applications such as *in vivo* dosimetry and for alternative detectors such as optically stimulated luminescence detectors (OSLD) that are becoming increasingly popular for dosimetry audits.^(^ ^10^ ^)^

## II. MATERIALS AND METHODS

Three methods of TLD calibration were investigated using the Nucletron V2 source (part no. 105.002, Nucletron B.V., Veenendaal, The Netherlands). For each method we first describe the technique used to calculate the dwell time at the defined point to deliver the prescribed dose. We then describe how we confirmed the calculation through measurement with an ionization chamber before proceeding to irradiate the TLDs. The fourth method describes the irradiation procedure using a 6 MV linac. In all cases, the aim was to deliver 1 Gy to the TLDs, as this was the dose that was prescribed for the Brachytherapy Audit. We present the results in terms of the results of the dosimetry audit. Uncertainty in the audit results are thus examined in relation to the uncertainty in the four alternative TLD calibration processes.

### A. Thermoluminescent dosimetry

All measurements were made with 6×1×1 mm (square) rods of lithium‐fluoride doped with magnesium and titanium (LiF:Mg,Ti) TLDs (Harshaw, type TLD‐100, Thermo Fisher Scientific Inc., Waltham, MA). TLD readout was performed using an automated TLD reader with hot nitrogen heating (Harshaw 5500; Thermo Fisher Scientific Inc.). Sensitivity factors were established for individual TLDs using standard irradiation, and the area under the glow curve for a temperature of 270°C was evaluated after a 10 s pre‐read anneal on 165°C. The TLDs were annealed at 400°C between two irradiations using a dedicated annealing oven (TLD4; S.E.M., Magill, South Australia). To account for the difference in response for each of the individual TLDs, a sensitivity correction was applied to each TLD as described by Meigooni et al.^(^ ^2^ ^)^

### B. Ionization chambers

All absorbed dose measurements made with the ${\;}^{192}\text{Ir}$ source (Methods 1–3) used either a PTW 30010 (Methods 1 and 3) or PTW TW 30013 chamber (Method 2) with a PTW UNIDOS E electrometer (PTW Freiburg GmbH, Freiburg, Germany). Both chambers have an external PMMA wall of thickness 0.335 mm, an internal graphite wall of thickness 0.09 mm, with an active volume defined by a radius and half length 3.1 mm 11.5 mm, respectively. The PTW 30010 air kerma chamber calibration coefficient ($N_{K,ARP}$) for use with Ir‐192 was derived through interpolation of response in a ${\;}^{60}\text{Co}$ and a kilovoltage X‐ray beam at the Australian Radiation Protection and Nuclear Safety Agency (ARPANSA) laboratory, as previously described.^(^ ^11^ ^)^ The air kerma calibration coefficient for the waterproof (PTW 30013) chamber, which has the same dimensions as the 30010 chamber, was derived through direct comparison, by measurement of the air kerma rate (AKR), using the ${\;}^{192}\text{Ir}$ source and air‐kerma calibration jig described in Method 1.

### C. Source calibration

**Figure 1:**
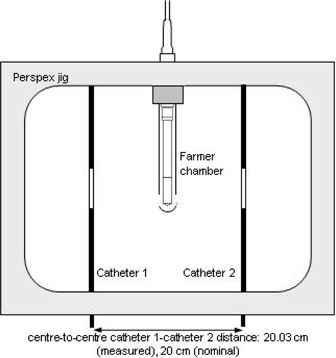
Schematic overhead view of the Nucletron‐type 077.211 jig and Farmer chamber (not to scale). This jig was used to determine the AKR of the source. A modified form of this jig was used for TLD calibration Methods 1 and 2, however the source‐to‐detector distance was 50 mm for these measurements (i.e., catheter 1‐catheter 2 distance was 100 mm)

The AKR of the ${\;}^{192}\text{Ir}$ source was determined 100 mm from the source using a Nucletron in‐air calibration jig (part no. 077.211, Nucletron B.V., Veenendaal, The Netherlands), the PTW 30010 chamber and the air kerma calibration coefficient determined at the National Physical Laboratory (NPL, $N_{K,\text{NPL}}$) in the UK.^(^ ^12^ ^)^ Details of this calibration procedure have been previously described;^(^ ^11^ ^–^ ^12^ ^)^ however, in contrast to the ARPANSA factor, the NPL factor takes into account a number of corrections such as scatter and non‐uniformity effects that would normally be applied in addition to the air kerma correction factor when following the recommendations of the International Atomic Energy Agency TecDoc 1274.^(^ ^13^ ^)^ To determine the AKR of the source, the chamber is placed in the jig assembly (Fig. 1). The ionization chamber reading is corrected for pressure and temperature effects, the NPL derived factor ($N_{K,\text{NPL}}$) applied, and the result is divided by the irradiation time. For the measurement of absorbed dose in water, however, the corrections included in the NPL calibration coefficient are not required. Hence, we used the ARPANSA‐derived factor ($N_{K,ARP}$) with the appropriate correction factors when the chamber was used to determine the absorbed dose.

### D. The brachytherapy audit

**Figure 2:**
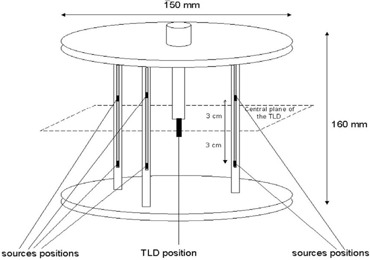
Water phantom used in the Australian Brachytherapy Audit. This design is based on the work of Roué et al.^(^ ^14^ ^)^

The design of the Brachytherapy Audit phantom is based on that of Roué et al.^(^ ^14^ ^)^ and is shown in Fig 2. The TLDs were placed in a PMMA holder in the center of a cylindrical, water‐filled phantom. The central TLD column is surrounded by three channels, at a distance of 50 mm from the central column that contain catheters that can be connected to the HDR unit. Each of the seven centers that participated in the Brachytherapy Audit was asked to deliver 1 Gy to 3 TLDs placed in the center of the phantom. The phantom was irradiated with three sets of TLDs; hence, each audit produced results for nine TLDs. We report the results of the audit as the ratio of the average TLD site reading (${\text{TLD}}_{A}$) divided by the average TLD calibration reading (${\text{TLD}}_{C}$).

#### E.1 Calibration Method 1: irradiation with ${\;}^{192}Ir$ 8 in air

The first method required the TLDs to be irradiated using an air‐jig assembly similar to the Nucletron air‐jig assembly, but with the additional feature of variable source–catheter distance. The TLDs were placed in a PMMA holder replacing the ionization chamber. The central axis of the catheters was placed at 50 mm from the central axis of the TLDs. For each irradiation, a set of three TLDs were irradiated by sending the source down each of the catheters so that it dwelled at a point 50 mm perpendicular to the central TLD to deliver a dose of 0.5 Gy (hence a total of 1 Gy was delivered from both catheters). The catheters in the jig have walls 0.28 mm thick and a correction factor for attenuation, ${\text{Att}}_{\text{cath}}$, of 1.0017 was included in the calculation of dwell times for the in‐air irradiations.^(^ ^11^ ^)^

The dwell time for each source position was calculated using the previously derived source AKR, applying the inverse square law, a factor to account for air attenuation and a factor to convert air kerma to absorbed dose. The IAEA TecDoc 1274^(^ ^13^ ^)^ Table XI indicates that air attenuation is approximately 0.1% per 100 mm, and so for a distance of 50 mm we have assumed this effect to be negligible. To convert air kerma to absorbed dose to water, we applied the ratio of mass energy absorption coefficients for water and air averaged over the ${\;}^{192}\text{Ir}$ spectrum (1.11).^(^ ^15^ ^)^

The wall thickness of the PMMA TLD holder was 2.7 mm. The attenuation factor for this holder, ${\text{Att}}_{\text{Hold}}$, was estimated by measuring the TLD response with increasing thicknesses of acrylic (in the form of cylinders mounted on the holder), and extrapolating the responses back to zero thickness. The attenuation was calculated from the ratio of the TLD response in the holder and the extrapolated response at zero thickness. Using this method, the attenuation of the holder was estimated to be 1.3% and the dwell times increased accordingly.

Prior to irradiating the TLDs, the dwell time calculation was confirmed through measurements made using the ionization chamber and catheter arrangement shown in Fig. 1, but with the ionization chamber placed 50 mm from the source. The absorbed dose was determined from the ionization chamber reading $M_{u}$ (corrected for pressure, temperature, and electrometer effects, but not for polarity effects or recombination) as follows:

(1) $$D_{w}(\text{in}\;\text{air})=M_{u}N_{K,ARP}{\left(\frac{{\bar{\mu }}_{tr}}{\rho }\right)}_{a}^{w}k_{n}$$

where $N_{K,ARP}$ is the ARPANSA derived air kerma chamber factor $(4.890\times 10^{7}\;Gy/C),\;{\left(\frac{{\bar{\mu }}_{tr}}{\rho }\right)}_{a}^{w}$ is the ratio of mass energy absorption coefficients (1.11^(^ ^15^ ^)^, and $k_{n}$ is the non‐uniformity correction factor which accounts for the nonuniform fluence across the chamber as a result of the steep dose gradient. We used the methodology of Bielajew^(^ ^13^ ^,^ ^16^ ^)^ as described by Butler et al.^(^ ^11^ ^)^ to determine a $k_{n}$ of 1.0351 for our chambers which have an active volume defined by a radius and half length 3.1 mm and 11.5 mm, respectively. Due to their small cross‐sectional area (1 × 1 mm), the effect of non‐uniformity in fluence across the TLDs was assumed to be negligible for all calibration methods.

(Note: In Methods 1–3 uncertainties due to the timer error and transit times were assessed and found to be less than 0.2% and, therefore, neglected.)

#### E.2 Calibration Method 2: irradiation with ${\;}^{192}\text{Ir}$ in water

The second method required the TLDs to be irradiated using the air‐jig assembly as described in Method 1 and similar to that shown in Fig. 1 with the TLDs and ionization chamber placed at 50 mm from the catheters and the whole assembly placed in a large (475×645×560 mm) water tank. For each irradiation, a set of three TLDs were irradiated by sending the source down each of the catheters so that it dwelled at a point 50 mm perpendicular to the central TLD to deliver a dose of 0.5 Gy (hence a total of 1 Gy was delivered from both catheters).

The dwell time for each source position was calculated using the previously derived source AKR, the TG‐43 formalism,^(^ ^9^ ^)^ and the parameters of Daskalov et al.,^(^ ^17^ ^)^ which at 50 mm perpendicular to the source, states the dose rate is $0.0446\;{\text{cGh}}^{-1}U^{-1}$, where U is defined as the air kerma strength and $U=1\;\mu \text{Gy}\;m^{2}h^{-1}$.

To confirm the dwell time calculation, measurements made with the ionization chamber were converted to absorbed dose using the methods of Tolli and Johansson^(^ ^18^ ^,^ ^19^ ^)^ who considered an adaptation of TRS 277^(^ ^20^ ^)^ using the high‐energy X‐ray formalism (which they refer to as the Bragg‐Gray method).

The modified Bragg‐Gray formalism, according to Tolli and Johansson, was defined as:

(2) $${\dot{D}}_{w}(P_{centre})=N_{D}(M_{u}/t){\bar{s}}_{w,a}p_{wall}p_{cel}p_{d}p_{n}$$

where $N_{D}$, the absorbed dose to air factor, was derived from the ARPANSA $N_{K,ARP}$ value, $N_{D}=N_{K}$(1‐g)$k_{\text{att}}$ $k_{m}$ (TRS 277^(^ ^20^ ^)^ and a value of g=0.003 (fraction of the energy of secondary charged particles lost to bremsstrahlung^(^ ^20^ ^)^ quoted for ${\;}^{60}\text{Co}$) has been assumed. A value for $k_{\text{att}}k_{m}$ for the PTW chamber is not listed in TRS 277 and so we used the value quoted in the PTW handbook (0.972) for the 30010 chamber to obtain an $N_{D}$ value of $4.74\times 10^{7}\;\text{Gy}/C$.

The stopping power ratio, $S_{w,a}$, used by Tolli and Johansson,^(^ ^18^ ^–^ ^19^ ^)^ was 1.137. For the perturbation due to the chamber wall $p_{\text{wall}}$, Tolli and Johansson quote a value of 1.018 for the A‐150 chamber and 1.001 (at 50 mm) for the graphite wall chamber. Ferreira et al.^(^ ^21^ ^)^ quotes a value for $A_{\text{wall}}$ (defined as the ionization chamber wall correction, which includes the PMMA cap) of 0.990 for the PTW chamber. We therefore used this value (0.990) in our calculations, though assumed an uncertainty of ±0.5%, as the chamber was placed directly in water and the cap not used.

According to Tolli and Johansson,^(^ ^19^ ^)^ a chamber with an aluminum central electrode over‐responds by approximately 2% at 50 mm from the source in water. Therefore, the proposed factor to account for the central electrode perturbation, $p_{\text{cel}}$, is 0.98.

$p_{d}$ is the correction factor for the displacement of the effective point of measurement. Using the formalism of Tolli and Johansson,^(^ ^18^ ^)^ the measurement point is at the center of the chamber. The non‐uniformity in fluence across the chamber is accounted for with the non‐uniformity factor $p_{n}$ (which is referred to as $k_{n}$ in Eq. (1)). During previous studies^(^ ^11^ ^)^ based on the work of Bielajew,^(^ ^16^ ^)^ at a distance of 50 mm from the source this has been determined to be equal to 1.0351. We have assumed an uncertainty of 1% rather than 0.4%, as this value was originally derived for measurements in air rather than water.

#### E.3 Calibration Method 3: irradiation with ${\;}^{192}Ir$ 8 in water using a modified version of the Brachytherapy Audit phantom

To irradiate the TLDs for this study, a new phantom was constructed using the same design as the Brachytherapy Audit phantom (Fig. 2); however, the central column was replaced with a PMMA column (wall thickness 1.5 mm for the PTW 30010 ionization chamber and 4.2 mm for the TLD holder) that provided a waterproof sleeve for either the PTW ionization chamber or the TLDs to be placed at the center of the phantom. Six dwell positions were programmed to deliver 1 Gy to the measurement point of the chamber (the center of the chamber) and the central TLD using a CT scan of the phantom and the Nucletron Plato V14.3.2 software which assumes a water‐equivalent homogenous medium. The software uses an AKR provided by the source manufacturer, which previously demonstrated agreement within ±1.5% of the AKR derived using the Nucletron air‐calibration jig and ionization chamber. The dwell positions were located 30 mm above and below the plane containing the chamber/TLDs, as shown in Fig 2; hence, the distance between each dwell position and the TLDs was 58.3 mm. These dwell positions are designed to produce a volume of uniform dose which permits some positional uncertainty in the dwell positions and still delivers the prescribed dose.

The catheters used for these measurements were the Nucletron 5F ProGuide catheters which have a wall thickness of 0.24 mm and were placed inside a PMMA rod with a wall thickness of 3.9 mm. The distance between the center of the catheter and the center of the central TLD was 50 mm which included 5.4 mm of plastic materials supporting the source and TLDs. Based on our previous work,^(^ ^11^ ^)^ we have assumed a transmission factor of 0.9965 for the PMMA material, and hence replacing 5.4 mm (of 50 mm of water) with PMMA would have a negligible effect on attenuation. In summary, no correction factors for the nonwater equivalence were included in the calculations.

To confirm the dwell time calculations, measurements made with the ionization chamber were converted to absorbed dose using the formalism and factors described in Method 2.

#### E.4 Calibration Method 4: irradiation with 6 MV X‐rays

**Figure 3:**
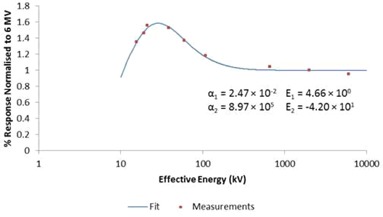
The energy response of the TLDs, ${\;}^{192}\text{Ir}$ relative to 6 MV, was determined by irradiating the TLDs in a range of energies and interpolating a value at 258 k V, the mean energy of ${\;}^{192}\text{Ir}$ in water at a depth of 50 mm.^(^ ^2^ ^)^ Curve fit is based on the methodology of Kron et al.^(^ ^1^ ^)^ and is derived from the measurements made with kV, ${\;}^{137}\text{Cs}$, and megavoltage X‐rays, and does not include the measurements made with the ${\;}^{192}\text{Ir}$ source.

A Varian (Pala Alto, CA) 600c 6 MV linear accelerator was used to irradiate eight TLDs on two separate occasions in solid water. The TLDs were placed at a depth of 100 mm in the solid water with a source‐to‐surface distance of 1000 mm, 100×100 mm field. Using standard methods, the number of monitor units required to deliver 1 Gy to the TLDs was calculated using the previously determined percentage depth dose at 100 mm. The TLDs were contained within the plane containing the central axis of an NE 2571 ionization chamber and perpendicular to the beam central axis. The formalism of TRS 398 and an absorbed dose to water chamber calibration coefficient (traceable to the Australian standard of absorbed dose for ${\;}^{60}\text{Co}$) was used to convert the ionization chamber readings to absorbed dose to water in the 6 MV beam. To derive an energy correction factor, the TLDs were irradiated in a range of X‐ray energies and a factor for ${\;}^{192}\text{Ir}$ was interpolated using the methodologies of Kron et al.^(^ ^1^ ^)^ assuming a mean energy of 258 keV^(^ ^2^ ^)^ for ${\;}^{192}\text{Ir}$ at a depth of 50 mm in water. The curve fit is based on the equation:

(3) $$R(E)=\{1-\text{exp}\left[-\alpha_{1}(E-E_{1})\right]\}\left[1+\alpha_{2}/{\left(E-E_{2}\right)}^{3}\right]$$

where $\alpha_{1}$ and $\alpha_{2}$ are two curve fit parameters (with values $0.0247{\text{eV}}^{-1}$ and $8.97\times 10^{5}\;{\text{eV}}^{-3}$, respectively) that determine the exponential fall‐off at low energies and the inverse cubic fall‐off towards higher energies, respectively. The other two curve fit parameters $E_{1}$ and $E_{2}$ (with values 4.66 keV and ‐42.0 keV, respectively) allow for an energy shift for the two components. All curve fit parameters were derived from measurements made with kilovoltage, ${\;}^{137}\text{Cs}$, and megavoltage X‐ray sources, and do not include the values derived from the measurements made with the ${\;}^{192}\text{Ir}$ source. The interpolated value was found to be 1.03 and we have assumed an uncertainty of 5% (1 SD) based on the recommendations of the AAPM TG‐43U1 report^(^ ^22^ ^)^ (Fig. 3).

## III. RESULTS

The results of the Brachytherapy Audit are stated as the ratio of the Audit site TLD (${\text{TLD}}_{A}$) readings compared with the readings from the TLD calibration set (${\text{TLD}}_{C}$). For Methods 1–3 there are two results: the first is based on the calculated dose (in all cases, the delivered dose was assumed to be 1 Gy based on the calculated time to deliver 1 Gy), and the second is based on the measured dose (i.e., the dose measured with the ionization chamber). The uncertainty in each Brachytherapy Audit result is therefore a combination (summed in quadrature) of the uncertainty in the TLD readings (${\text{TLD}}_{A}$ and ${\text{TLD}}_{C}$) and the uncertainties in either the calculated or measured dose. Each of the uncertainties associated with the calculated and (ionization chamber) measured dose is discussed in the Discussion section, with further details provided in the online supplementary material.

### A. TLD readings

At the audit sites, a total of nine TLDs were exposed to a nominal dose of 1 Gy. The standard deviation of the nine TLDs from each site was always within ±2%, and therefore, as we present the result of the average of nine readings, the assumed uncertainty in the audit site TLD reading is reduced by a factor of 3 (i.e., 0.7%). Similarly for each of the calibration methods described in this paper, a minimum of nine TLDs were irradiated and the standard uncertainty is again 0.7%.

#### A.1 Method 1: irradiation with ${\;}^{192}Ir$ 8 in air

The dose measured with the ionization chamber and the in‐air jig was 2.2% higher than the calculated dose (Table 1). Based on the in‐air calculated or measured dose, the results of the TLD audit indicate that, on average, centers delivered a dose 2% or 4% higher than prescribed respectively (Table 2).

**Table 1:** Values and uncertainty estimates (1 σ) for Method 1, irradiation in air. Dwell times were calculated to deliver 1 Gy. The measured dose was 1.022 Gy (i.e., 2.2% higher than predicted). The uncertainty in d is estimated to be 0.5 mm, resulting in a 2% uncertainty in the calculated absorbed dose. The total uncertainty is the sum in quadrature of all the components and does not include the uncertainty in the TLD reading (0.7%).

*Quantity*	*Value*	*Units*	*Uncertainty (%)*
*Calculation of* $D_{w,in\;air}$ *from AKR*			
AKR (at 1 m)	$8.85\times 10^{-6}$	$\text{Gy}\;s^{-1}$	1
Distance *d* in air	50	mm	2
Correction for catheter attenuation, $Att_{cath}$	1.0017		0.2
Correction for TLD holder attenuation, $Att_{Hold}$	1.013		2
Ratio of mass energy absorption coefficients, ${\left(\frac{{\bar{\mu }}_{tr}}{\rho }\right)}_{a}^{w}$	1.11		3
Dwell time for 1 Gy =	258.2	s	4%
$Att_{cath}\times Att_{Hold}/[AKR\times {\left(1000/d\right)}^{2}\times {\left(\frac{{\bar{\mu }}_{\sigma }}{\rho }\right)}_{a}^{w}]$			
*Measurement with ionisation chamber*			
$M_{u}$	$1.81\times 10^{-8}$	C	< 0.1
$k_{TP}k_{elec}$	1.006		0.1
$N_{K,ARP}$	$4.89\times 10^{7}$	$\text{Gy}\;C^{-1}$	1
Ratio of mass energy absorption coefficients, ${\left(\frac{{\bar{\mu }}_{tr}}{\rho }\right)}_{a}^{w}$	1.11		3
$k_{n}$	1.035		0.4
Distance *d* in air	50	mm	2
$D_{w,in\;air}=M_{u}\times k_{TP}k_{elec}\times N_{K,ARP}\times {\left(\frac{{\bar{\mu }}_{tr}}{\rho }\right)}_{a}^{w}\times k_{n}$	1.022	Gy	4%

**Table 2:** The results of the Brachytherapy Audit for the different TLD calibration methods. ‘Calculated’ values are derived from the AKR of the source. ‘Measured’ values are determined with an ionization chamber at the position of TLD irradiation.

*Center No*	*Method 1 (air, calc.)*	*Method 1 (measured)*	*Method 2 (water, calc.)*	*Method 2 (measured)*	*Method 3 (phantom, calc.)*	*Method 3 (phantom, measured)*	*Method 4, with energy correc. factor*
1	1.04	1.06	1.02	1.03	1.04	1.03	1.08
2	1.03	1.06	1.01	1.02	1.04	1.02	1.07
3	1.01	1.03	0.99	1.00	1.01	1.00	1.05
4	1.03	1.05	1.00	1.02	1.03	1.01	1.07
5	1.00	1.02	0.98	0.99	1.00	0.99	1.04
6	1.01	1.03	0.99	1.00	1.01	1.00	1.05
7	0.99	1.02	0.97	0.98	1.00	0.98	1.03
Average	1.02	1.04	0.99	1.01	1.02	1.00	1.06

#### A.2 Method 2: irradiation with ${\;}^{192}Ir$ 8 in water

The measured dose was 1.1% higher than the calculated dose (Table 3). Based on the calculated and measured dose, the results of the TLD audit indicate that, on average, centers delivered a dose 1% lower or 1% higher than the prescribed dose respectively (Table 2).

**Table 3:** Values and uncertainty estimates (1 σ) for Method 2, irradiation in the Nucletron jig in water. Dwell times were calculated to deliver 1 Gy. The measured dose was 1.011 Gy (i.e., 1.1% higher than predicted). The uncertainty in d is estimated to be 0.5 mm, resulting in a 2% uncertainty in the calculated absorbed dose.

*Quantity*	*Value*	*Units*	*Uncertainty in* $D_{w}$ *(%)*
*Calculation of* $D_{w}$ *from AKR*			
AKR (at 1 m)	$8.85\times 10^{-6}$	$\text{Gy}\;s^{-1}$	1
Distance *d* in water	50	mm	2
Dose at 50 mm per unit air kerma at 1 m, *R*	446		2
Dwell time for 1 Gy=1/(R×AKR)	253.3	s	3%
*Measurement with ionisation chamber*			
$M_{u}$	$1.81\times 10^{-8}$	C	< 0.1
$k_{TP}k_{elec}$	1.006		0.1
$N_{D}$ calculated by TRS‐277 from $N_{k,Ir-192}$	$4.74\times 10^{7}$	$\text{Gy}\;C^{-1}$	1
Average stopping power ratio, water to air, *s*	1.137		0.3
$p_{Wall}$	0.99		0.5
$p_{cel}$	0.98		0.5
$p_{n}(=k_{n})$	1.035		1
Distance *d* in water	50	mm	2
$D_{w}=M_{u}\times k_{TP}k_{elec}\times N_{D}\times s\times p_{nwall}\times p_{ncel}\times p_{n}$	1.011	Gy	3%

#### A.3 Method 3: irradiation with ${\;}^{192}Ir$ 8 in water using a modified version of the Brachytherapy Audit phantom

Using Method 3, the measured dose was 1.5% lower than the calculated dose (Table 4). Based on the measured dose, the results of the TLD audit indicate that, on average, centers delivered the dose as prescribed (Table 2).

**Table 4:** Values and uncertainty estimates (1 σ) for Method 3, irradiation in a modified audit phantom in water. Dwell times were calculated to deliver 1 Gy using the treatment planning system. The measured dose was 0.985 Gy (i.e., 1.5% lower than predicted). The uncertainty in d is estimated to be 0.3 mm, resulting in a 1% uncertainty in the calculated absorbed dose. The total uncertainty is the sum in quadrature of all the components and does not include the uncertainty in the TLD reading (0.7%).

*Quantity*	*Value*	*Units*	*Uncertainty in* $D_{w}$ *(%)*
*Calculation of* $D_{w}$ *from AKR and phantom CT by treatment planning system*			
AKR (at 1 m)	$5.31\times 10^{-6}$	$\text{Gy}\;s^{-1}$	1.5
Distance *d* in water	58.3	mm	1
Dose at 58.3 mm per unit air kerma at 1 m, *R*	317.95		2
Dwell time for 1 Gy=1/ (R×AKR)	592.8	s	3%
*Measurement with ionisation chamber*			
$M_{u}$	$1.79\times 10^{-8}$	C	<0.1
$k_{TP}k_{elec}$	1.006		0.1
$N_{D}$ calculated by TRS‐277 from $N_{K,Ir-192}$	$4.74\times 10^{7}$	$\text{Gy}\;C^{-1}$	1
Average stopping power ratio, water to air, *s*	1.137		0.3
$p_{Wall}$	0.99		0.5
$p_{cel}$	0.98		0.5
$p_{n}(=k_{n})$	1.035		1
Distance *d* in water	58.3	mm	1
$D_{w,}=M_{u}\times k_{TP}k_{elec}\times N_{D}\times s\times P_{nwall}\times p_{ncel}\times p_{n}$	0.985	Gy	2%

#### A.4 Method 4: irradiation with 6 MV X‐rays

Using Method 4, the results of the TLD audit indicate that, on average, centers delivered a dose 6% higher than the prescribed dose (Table 2).

## IV. DISCUSSION

We have described four different methods of TLD calibration for applications involving high dose rate brachytherapy. For Methods 1–3, prior to TLD irradiation we calculated the amount of time the source should rest at its dwell position to deliver the required dose and we confirmed this calculation by direct measurement. The results from the Brachytherapy Audit therefore carry an uncertainty which is the sum in quadrature of the uncertainty in the TLD readings (${\text{TLD}}_{A}$ and ${\text{TLD}}_{C}$, each 0.7%) and the uncertainties in either the calculated or measured dose. Accurate dosimetry for ${\;}^{192}\text{Ir}$ is challenging in the absence of a suitable formalism and a dose‐to‐water primary standard, as such, each of the methods of calculation and measurement carry a wide range of uncertainties, many of which have been estimated, which has resulted in an overall uncertainty in the results of the Brachytherapy Audit. The estimated standard uncertainties are summarized in Tables 1,3,4,5 and discussed in detail below.

**Table 5:** Values and uncertainty estimates (1 SD) for Method 4, irradiation in 6 MV photons. The total uncertainty is the sum of all errors taken in quadrature and does not include the uncertainty in the TLD reading (0.7%).

		*Value*	*Units*	*Standard Uncertainty (%)*
Measurement	Measured dose (TRS 398)	0.999	Gy	1.5
	SSD/Depth	1000/100	mm	0.6
	Energy Correction Factor	1.03		5
	Total			5%

### A. Method 1: irradiation with ${\;}^{192}\text{Ir}$ in air

The AKR of the source was determined using the NPL chamber factor ($N_{K,\text{NPL}}$ ^(^ ^12^ ^)^ which has a standard uncertainty of 0.55%. Measurement of AKR is relatively simple as long as the chamber and calibration jig are placed more than 1 meter from scatter surfaces such as walls and floor. We therefore have a high level of confidence in this factor, but have assumed a standard uncertainty of 1% to take into account that we were not using the original calibration jig. The TLDs were irradiated using a modified jig that allowed irradiation at a distance of 50 mm from the source rather than 100 mm in the standard calibration jig, to be consistent with the geometry used in the Brachytherapy Audit. Methods 1 and 2 introduced an uncertainty in dose of 2% due to the geometric uncertainty (0.5 mm) in the distance of the source to the measurement point of the TLD and chamber.

The TLDs were placed in a PMMA TLD holder. The attenuation of the irradiating source due to this holder was estimated by extrapolation of data measured with varying thickness of material. However, it is not clear that this method is valid as the quality of the radiation reaching the TLDs for a range of material thickness is likely to change considerably due to rapid absorption of the low‐energy components of the primary and scattered radiation. It is difficult to estimate the uncertainty in this factor, and this will be the subject of future studies using Monte Carlo methods. For now we have assumed an uncertainty of 2% to incorporate measurement uncertainty and uncertainty in the validity of the method used to derive this factor.

For all ionization chamber measurements made with the ${\;}^{192}\text{Ir}$ source, polarity and recombination corrections were not included in any of the calibration coefficients quoted in this paper. The recombination correction for Farmer chambers is small for the air kerma rates used for these measurements (approximately 3.5 mGy/s at 50 mm) and has been ignored.^(^ ^11^ ^)^ The polarity correction is also expected to be small and an uncertainty of 0.2% is included in the value of $N_{k}$ to take this into account.^(^ ^11^ ^)^

In both the measurement and calculation of dwell times, we have used the ratio of mass energy absorption coefficients for water and air averaged over the ${\;}^{192}\text{Ir}$ spectrum (1.11 with standard uncertainty 3%) to determine the absorbed dose to water. When TLDs calibrated in this way are used in water, the spectrum incident on the TLD will include more low‐energy photons due to scatter in the water, to which the TLD may overrespond. Based on the energy response data shown in Fig. 3, the TLD energy response factor for a mean energy of 397 keV (the mean energy of ${\;}^{192}\text{Ir}$ in air^(^ ^13^ ^)^ relative to 6 MV is 1.01, suggesting a 1% over‐response. As the TLDs were placed in a PMMA holder 2.7 mm thick, we would expect this over‐response to be less than 1%. This could however, potentially explain why the audit results were higher than expected (2% or 4% based on the in‐air calculated or measured dose, respectively). Furthermore, the TLDs were irradiated in a plane perpendicular to the source, which is in contrast to the geometry for the audit TLDs which were contained in a plane 30 mm above and below the source. Such variations in geometry (which also apply to Methods 2 and 4) will again produce differences in the spectrum leading to variations in the TLD response. Based on the work of Karaiskos et al.,^(^ ^6^ ^)^ we have assumed the angular response of the TLDs to be negligible at less than 1%. A standard uncertainty of 3% has been included in the uncertainty analysis to account for these uncertainties. It is not clear, however, if the value we have used for the ratio of mass energy absorption coefficients for water and air is correct in this setting, and we therefore have the least confidence in this method of calibration due to uncertainties in the factor used to determine the absorbed dose to water from the calculation/measurement of air kerma and the attenuation factor of the TLD holder. In addition there are a number of factors that are common to both the calculation and measurement methods limiting the independence of these methodologies. The method, however, is reasonably simple to use once a suitable calibration jig has been constructed and a chamber calibration coefficient determined. In summary, the total uncertainty budget for Method 1 (including the ${\text{TLD}}_{A}$ and ${\text{TLD}}_{C}$ readings) is 4% (0.7%, 0.7%, and 4% summed in quadrature) when the dose to the calibration TLDs is based on calculated or measured dose.

### B. Method 2: irradiation with ${\;}^{192}\text{Ir}$ in water

Calculation of dwell times for the in‐water measurement relied on the data provided by Daskalov et al.^(^ ^17^ ^)^ and the previously measured air kerma rate based on the NPL‐derived chamber calibration coefficient. The data from the Daskalov study are based on Monte Carlo calculations and have not been verified experimentally, but are supported by previous calculations and measurements with similar sources. Daskalov and colleagues state that their calculations agree with measurements to within their experimental error of 5% within 70 mm of the source, and we have taken the standard uncertainty to be approximately half this value at 50 mm.

As there is no standard formalism for measurement of absorbed dose to water using a Farmer type chamber, we used the modified Bragg‐Gray formalism according to Tolli and Johansson^(^ ^19^ ^)^ which uses an absorbed dose‐to‐air chamber factor rather than an air kerma‐based factor. To derive a value of $N_{D}$ from $N_{K}$ we assumed a value of g=0.003 based on published values for ${\;}^{60}\text{Co}$.^(^ ^20^ ^)^ However, it is expected this value will be less for ${\;}^{192}\text{Ir}$, but the uncertainty in this value will be negligible compared with other factors. For the remaining factors, we used a combination of values published by Tolli and Johansson,^(^ ^19^ ^)^ Ferreira et al.,^(^ ^21^ ^)^ Bielajew,^(^ ^16^ ^)^ and Butler et al.^(^ ^11^ ^)^ and assumed their published uncertainty for each of these factors, resulting in an overall uncertainty as shown in Table 3. In addition to the uncertainty in deriving absorbed dose from the ionization chamber reading, we must also include the uncertainty in dose due to uncertainty in distance from the source (2%), as described in Method 1.

This method of TLD calibration was the most difficult of all methods to perform and is least practical for regular TLD calibration as it required careful placement of the delicate in‐air jig into the large water tank. It was, however, possible to minimize the amount of non‐water equivalent material in the irradiation field. The method of calculation used the Daskalov tables^(^ ^17^ ^)^ which contain the same data used by the Nucletron brachytherapy planning computers used by most of the centers in the audit. The calculation method, therefore, does carry some degree of interdependence (e.g., should the Daskalov data carry a systematic error, this would not be detected through the audit if we relied on the calculations of Method 2). The close agreement of the measured and calculated results (within 1.1%) confirm our confidence in this method and if we assume an overall uncertainty of 3% (including the 0.7% uncertainty in TLD readings) for this method of calibration, we can say that all sites that participated in the audit irradiated their TLDs to 1 Gy within the expected uncertainty. In summary, the total uncertainty budget for Method 2 (including the ${\text{TLD}}_{A}$ and ${\text{TLD}}_{C}$ readings) is 3% (0.7%, 0.7%, and 3% summed in quadrature) when the dose to the calibration TLDs is based on calculated or measured dose.

### C. Method 3: irradiation with ${\;}^{192}\text{Ir}$ in water using a modified version of the Brachytherapy Audit phantom

The uncertainties described in Method 2 mostly apply to Method 3. There are, however, three major differences (summarized in Table 5). As Method 3 uses a rigid phantom there is less uncertainty in the distance of the source from the TLDs and this distance could be measured with the aid of the CT scanner with a pixel width of 0.8 mm. Uncertainty in source position was therefore due only to the uncertainty of the source (diameter 0.9 mm) within the catheter (inner diameter 1.2 mm). The dwell positions used for this method were identical to those used in the Brachytherapy Audit measurements and therefore we would expect any uncertainties due to change in TLD response due to source‐detector distance to be minimized. The phantom, however, needed to be constructed with a larger amount of non‐water equivalent material to allow interchangeable placement of the chamber and TLDs at the measurement point. This introduces an additional uncertainty, which we have estimated to be less than 0.5%. This will be verified in future studies using Monte Carlo methods. The difference between the calculated and measured dose (1.5%) may be attributed to differences between the AKR stated by the source manufacturer (and used by the planning computer to calculate the dwell times) and the AKR that had been measured on a previous occasion with the ionization chamber. The audit results for this method were, not surprisingly, very similar to Method 2 and, therefore, if we assume an overall uncertainty of 3% for the calculation method and 2% for the measurement method (including the 0.7% uncertainty in TLD readings) for this method of calibration, we can say that all sites that participated in the audit irradiated their TLDs to 1 Gy within the expected uncertainty. In summary, the total uncertainty budget for Method 3 (including the ${\text{TLD}}_{A}$ and ${\text{TLD}}_{C}$ readings) is 3% or 2% (0.7%, 0.7%, and 3%/2% summed in quadrature), respectively, when the dose to the calibration TLDs is based on calculated or measured dose.

This method was very simple to use, though irradiation time can be long (more than 8 minutes for a decayed source).

### C. Method 4: irradiation with 6 MV X‐rays

Irradiating the TLDs using a clinical linac is relatively quick, the dosimetry has a high level of confidence due to the availability of internationally accepted data, and geometric uncertainties are minimal. Using a high‐energy clinical linac with a relatively flat beam and minimal uncertainties from using water‐equivalent materials provided a high level of confidence in the dose delivered to the TLDs. Uncertainties in measured dose relate only to the correction factors applied to the ionization chamber reading and a small positional uncertainty relating the measurement point of the ionization chamber to the TLDs (< 1 mm). The major disadvantage of this method, however, is the introduction of the energy correction factor. The energy correction factor for TLD‐100 LiF:Mg,Ti measured by Meigooni et al.^(^ ^2^ ^)^ was 1.045, with a standard uncertainty of 3%, at a depth of 5.32 cm in polystyrene. This factor varied by up to 8.5% when the depth of overlying phantom material ranged from 10 to 100 mm, and this variation was assumed to be related to changes in the ${\;}^{192}\text{Ir}$ spectrum with depth. This variation of TLD response with energy, however, was challenged by Thomason and Higgins^(^ ^5^ ^)^ who determined the energy response was negligible (within experimental uncertainty) and challenged the experimental methods used by Meigooni to explain their difference in results. Over decades, many authors have quoted an energy correction factor for LiF:Mg,Ti interpolated from measurements made with kilovoltage units (which carry a larger degree of uncertainty in dose, approximately 5%) and megavoltage therapy linacs, ${\;}^{60}\text{Co}$ or ${\;}^{137}\text{Cs}$ sources.^(^ ^1^ ^–^ ^3^ ^,^ ^5^ ^,^ ^23^ ^–^ ^28^ ^)^ Each of these investigators have used a variety of narrow beam and heavily or lightly filtered kilovoltage sources and a range of methods to determine mean energy of the irradiating source. Based on our work in deriving the energy factor of 1.03 (from the data shown in Fig. 3), we have assumed ${\;}^{192}\text{Ir}$ has a mean energy of 258 keV^(^ ^2^ ^)^ at a depth of 50 mm in water. To compare our work with Meigooni et al.^(^ ^2^ ^)^ and assume an average photon energy of 337 keV at 10 mm and 221 keV at 100 mm (as suggested by Meigooni and colleagues), the energy correction factors would be 1.02 and 1.04, respectively (i.e., a variation of less than 3% over a radial distance of 100 mm), which is consistent with the findings of Karaiskos et al.^(^ ^6^ ^)^ and considerably less than up to 8.5% as suggested by Meigooni et al.^(^ ^2^ ^)^

An alternative to using a 6 MV linac would be the use of a ${\;}^{60}\text{Co}$ unit. The average energy of ${\;}^{60}\text{Co}$ (1.25 MeV) is closer to ${\;}^{192}\text{Ir}$ and therefore potentially minimizes the uncertainty in interpolation over a shorter energy range. Based on the data shown in Fig. 3 however, the energy response of ${\;}^{192}\text{Ir}$ relative to ${\;}^{60}\text{Co}$ is still approximately 3%. We did not have access to a therapy ${\;}^{60}\text{Co}$ unit for this work. A cell irradiator was made available for the data point shown in Fig. 3, however the dosimetry for this unit does not carry the same level of accuracy as a therapy unit and therefore is not suitable for calibration of TLDs.

Differences in heating (temperature profiles) and annealing processes may also have an effect on TLD response^(^ ^3^ ^)^ and may also partly explain differences in values reported in the literature. Furthermore, whilst we may be comparing TLDs with identical chemical composition, the detectors may vary in doping, shape, and size, leading to variations in response due to volume averaging, detector self‐absorption, and perturbation effects.^(^ ^25^ ^)^ Unfortunately, deriving the response from interpolation in a range of X‐ray energies may also carry a large degree of uncertainty and this will most likely be the largest source of error in the overall uncertainty in TLD readings. In line with AAPM TG‐43U1 recommendations,^(^ ^22^ ^)^ we have assumed a standard error of ±5% in our derived energy correction factor for the reasons stated above and because of the limited data in the energy range between our highest kilovoltage source (100 keV) and our reference 6 MV linac. This factor, however, cannot have a value less than 1.00 and so the standard error is more likely to be ‐3% to +5% Based on this method, however, it would appear that all centers delivered a dose higher than expected (Table 2). Although each of the three TLD calibration methods that use the ${\;}^{192}\text{Ir}$ source have some common factors in measurement or calculation (AKR for example) and there does not exist a universally accepted dosimetry formalism, we suggest that the source of discrepancy between the ${\;}^{192}\text{Ir}$ methods and the 6 MV method may be due to some underlying source of systematic uncertainty in the energy correction factor used with Method 4 when applied to the phantom measurements. It should be noted that if the energy correction factor of 1.03 had not been applied, then the values shown in the final column of Table 2 would have been 3% higher (i.e., this would indicate centers delivered a dose 9% higher than predicted). In summary, the total uncertainty budget for Method 4 (including the ${\text{TLD}}_{A}$ and ${\text{TLD}}_{C}$ readings) is 5% (0.7%, 0.7%, 5% summed in quadrature) when the dose to the calibration TLDs is based on measured dose. This uncertainty is dominated by the 5% uncertainty in the energy correction factor, and therefore this method may be more suitable for use with detectors with a smaller energy response such as OSLDs.^(^ ^29^ ^)^

### D. The Brachytherapy Audit results

The results of the Brachytherapy Audit are summarized in Table 2. Tolerance limits set by previous similar audits^(^ ^14^ ^,^ ^24^ ^)^ were defined as ‘optimal’ if within 1.5 SD and ‘within tolerance’ if within 2 SD. The combined standard uncertainty for the European^(^ ^14^ ^)^ and Brazilian audits^(^ ^24^ ^)^ was 6.54% (2 SD) and 2.8% (k=2.03, confidence interval 95.7%), respectively. This translates to optimal levels of ≤5% and ≤3%, and tolerance levels of 5%–7% and 3%–6%, respectively. Based on the TLD methods presented in this paper, optimal and tolerance levels would range from ≤3% and 3%–4% (using TLD calibration Method 3, based on measured dose) to ≤7.5% and 7.5%–10% (for Method 4). Using this criterion, we can conclude that all centers participating in the audit were within the optimal level.

## V. CONCLUSIONS

We present four methods for calibration of TLDs for use with high‐dose rate ${\;}^{192}\text{Ir}$ applications. Using a clinical linear accelerator is convenient, the dosimetry is well understood, and several TLDs can be irradiated in a short period of time. The major disadvantage of this method is the uncertainty in the energy correction factor, which is dependent on a range of measurement conditions and TLD characteristics. The uncertainty in this method was the highest of all methods investigated (±5%) and all dose measurements using this method were consistently higher than the methods using the ${\;}^{192}\text{Ir}$ source. Calibrating TLDs with the ${\;}^{192}\text{Ir}$ source requires careful measurement setup due to the high dose gradients surrounding the source. Consensus documents providing rigorous review of the correction factors applied to commonly used ionization chambers for measurement of absorbed dose do not exist, leading to some uncertainty in measurement of dose. In each of the methods using the ${\;}^{192}\text{Ir}$ source, it was necessary to use a common source of data; however, all three methods resulted in similar audit results. Method 3 used a phantom very similar in design to the Audit phantom (which was the purpose of this work) and hence a similar geometry in terms of distance between source and TLD. In addition, it used similar amounts of water and phantom material, and it provided minimal uncertainty in source — TLD distance and the overall uncertainty was lowest (2%). Method 3 is, therefore, our chosen method for presentation of the results of the Brachytherapy Audit.

## ACKNOWLEDGMENTS

The authors would like to acknowledge Dr Ivaldo Ferreira, formerly of the EQUAL‐ESTRO Laboratory, Villejuif, France for his advice in carrying out these measurements. We would also like to thank Dr Jack Venselaar for sharing the details of the European audit. We acknowledge the excellent work of Peter Pinder and his team at the Peter MacCallum Cancer Centre for their expertise in manufacturing the high precision phantoms. We are also most grateful to The Wesley Cancer Care Centre (Premion), The Peter MacCallum Cancer Centre, The University of Newcastle, The Calvary Mater Hospital, Nucletron Australia, Global Medical Solutions, Varian Oncology Systems for their generous support of this project.

## Supporting information

Supplementary Material
