# Supplementary material for: Comparison of TLD calibration methods for 192Ir dosimetry
Source: J Appl Clin Med Phys. 2013 Jan 7;14(1):258–72. doi: 10.1120/jacmp.v14i1.4037 (PMC5714053; doi:10.1120/jacmp.v14i1.4037)
Supplement: Supplementary file 1 — Supplementary Material [file ACM2-14-258-s001.doc]

**Supplementary on-line material:**

**Additional details of the TLD calibration methods and uncertainties**

These supplementary tables give the full calculations of the TLD calibration coefficients and uncertainties for each method. The uncertainties have been estimated at the one-sigma level (standard uncertainties). In each case the degrees of freedom are thought to be large enough to have a negligible affect on the final result, and hence are not included. Uncertainties due to source transit times and timer error have been found to be negligible. Uncertainties have been retained to two significant figures for clarity, although in most cases only one figure is justified.

**Table A1.** Summary of the methods of TLD calibration and uncertainties in the TLD calibration coefficients determined by each method.

|  | Method 1 | | Method 2 | | Method 3 | | Method 4 |
| --- | --- | --- | --- | --- | --- | --- | --- |
| Source | Ir-192 | | Ir-192 | | Ir-192 | | 6 MV linac |
| Jig | Nucletron | | Nucletron | | Audit phantom | | None |
| Medium | Air | | Water | | Water | | Solid water |
| Distance | 50 mm | | 50 mm | | 58.3 mm | | 1100 mm |
| Dosimetry method | 1A | 1B | 2A | 2B | 3A | 3B | 4 |
| Dosimetry | Calculated from AKR | Ion chamber | Calculated from AKR | Ion chamber | Calculated from AKR | Ion chamber | Ion chamber |
| Quantity | *D_w,in air_* | *D_w,in air_* | *D_w_* | *D_w_* | *D_w_* | *D_w_* | *D_w_* |
| TLD_cc_* (Gy/nC) | 2.81×10^-4^ | 2.87×10^-4^ | 2.75×10^-4^ | 2.79×10^-4^ | 2.81×10^-4^ | 2.77×10^-4^ | 2.92×10^-4^ |
| Uncertainty (%) | 4.3 | 3.8 | 3.1 | 2.8 | 2.8 | 2.1 | 5 |

*The calibration coefficient of the TLD, where nC is the unit of the raw reading from the photomultiplier when the TLD was read out.

**Table A2: Method 1A absorbed dose to water calculated from reference air kerma rate for the Nucletron jig in air**

| Quantity | Symbol | Value | Uncertainty u | Units | Type | Standard u in *D_w,in air_* (%) |
| --- | --- | --- | --- | --- | --- | --- |
| Reference air kerma rate (at 1 m) | *AKR* | 8.85 × 10^-6^ | 9 × 10^-8^ | Gy s^-1^ | B | 1 |
| Source-TLD distance | *d* | 50 | 0.5 | mm | B | 2 |
| Correction for catheter attenuation | *Att_cath_* | 1.002 | 0.002 | - | B | 0.2 |
| Correction for TLD holder attenuation | *Att_hold_* | 1.013 | 0.007 | - | B | 2 |
| Ratio of mass energy absorption coefficients | *(μ_tr_/ρ)_w,air_* | 1.11 | 0.033 | - | B | 3 |
| Exposure time per dwell position | *t* | 129.1 | <0.1 | s | B | <0.1 |
| Number of dwell positions | *n* | 2 | 0 | - | B | 0 |
| (Subtotal) Absorbed dose to water (in air) | *D_w, in air_* | 1.000 |  | Gy |  | 4.2 |
| TLD readout (average of 9 rods) |  | 3559.7 | 24.9 | nC | A | 0.7 |
| TLD calibration coefficient |  | 2.81 × 10^-4^ |  | Gy/nC |  | 4.3 |

**Table A3: Method 1B absorbed dose to water calculated from ionisation chamber in Nucletron jig in air**

| Quantity | Symbol | Value | Uncertainty u | Units | Type | Standard u in D_w,in air_ (%) |
| --- | --- | --- | --- | --- | --- | --- |
| Ion chamber reading at 50 mm in air, cap on | *M_u_* | 1.81 × 10^-8^ | 2 × 10^-11^ | C | A | 0.1 |
| Correction for temperature, pressure, electrometer | *k_TP_.k_elec_* | 1.006 | 0.0005 | - | B | 0.1 |
| Positioning of ion chamber relative to TLDs | *-* | - | 0.5 | mm | B | 2 |
| Ion chamber calibration coefficient from ARPANSA | *N_K,Ir-192_* | 4.89 × 10^7^ | 5 × 10^5^ | Gy/C | B | 1 |
| Correction for non-uniformity at 50 mm in air | *k_n_* | 1.035 | 0.004 | - | B | 0.4 |
| Ratio of mass energy absorption coefficients | *(μ_tr_/ρ)_w,air_* | 1.11 | 0.033 | - | B | 3 |
| (Subtotal) Absorbed dose to water (in air) | *D_w, in air_* | 1.022 |  | Gy |  | 3.7 |
| TLD readout (average of 9 rods) |  | 3559.7 | 24.9 | nC | A | 0.7 |
| TLD calibration coefficient |  | 2.87 × 10^-4^ |  | Gy/nC |  | 3.8 |

**Table A4: Method 2A absorbed dose to water calculated from reference air kerma rate for the Nucletron jig in water**

| Quantity | Symbol | Value | Uncertainty u | Units | Type | Standard u in D_w_ (%) |
| --- | --- | --- | --- | --- | --- | --- |
| Reference air kerma rate (at 1 m) | *AKR* | 8.85 × 10^-6^ | 9 × 10^-8^ | Gy s^-1^ | B | 1 |
| Source-TLD distance | *-* | - | 0.5 | mm | B | 2 |
| Daskalov ratio* | *k_Dask_* | 446 | - | - | B | 2 |
| Exposure time per dwell position | *t* | 126.7 | <0.1 | s | B | <0.1 |
| Number of dwell positions | *n* | 2 | 0 | - | B | 0 |
| (Subtotal) Absorbed dose to water (in air) | *D_w_* | 1.000 |  | Gy |  | 3.0 |
| TLD readout (average of 9 rods) |  | 3638.9 | 25.5 | nC | A | 0.7 |
| TLD calibration coefficient |  | 2.75 × 10^-4^ |  | Gy/nC |  | 3.1 |

*The ratio of absorbed dose to water rate at the given depth to the reference air kerma rate at 1 m, as calculated by Daskalov [16].

**Table A5: Method 2B absorbed dose to water calculated from ionisation chamber in Nucletron jig in water**

| Quantity | Symbol | Value | Uncertainty u | Units | Type | Standard u in D_w_ (%) |
| --- | --- | --- | --- | --- | --- | --- |
| Ion chamber reading at 50 mm in water, cap off | *M_u_* | 1.86 × 10^-8^ | 2 × 10^-11^ | C | A | 0.1 |
| Correction for temperature, pressure, electrometer | *k_TP_.k_elec_* | 1.006 | 0.001 | - | B | 0.1 |
| Positioning of ion chamber relative to TLDs | *d* | 50 | 0.5 | mm | B | 2 |
| Ion chamber calibration coefficient from ARPANSA | *N_K,Ir-192_* | 4.89 × 10^7^ | 4.89 × 10^5^ | Gy/C | B | 1 |
| TRS-277 corrections *k_att_.k_m_* | *k_att_.k_m_* | 0.972 | 0.002 | - | B | 0.2 |
| Correction for bremstrahlung loss | 1-*g* | 0.997 | 0.001 | - | B | 0.1 |
| Average stopping power ratio | *s_w,air_* | 1.137 | 0.003 | - | B | 0.3 |
| Wall correction | *p_wall_* | 0.99 | 0.005 | - | B | 0.5 |
| Central electrode correciton | *p_cel_* | 0.98 | 0.005 | - | B | 0.5 |
| Correction for non-uniformity at 50 mm in water | *k_n_* | 1.035 | 0.010 | - | B | 1 |
| (Subtotal) Absorbed dose to water | *D_w_* | 1.011 |  | Gy |  | 2.7 |
| TLD readout (average of 9 rods) |  | 3638.9 | 25.5 | nC | A | 0.7 |
| TLD calibration coefficient |  | 2.79 × 10^-4^ |  | Gy/nC |  | 2.8 |

*The ratio of absorbed dose to water rate at the given depth to the reference air kerma rate at 1 m, as calculated by the treatment planning system, based on the Daskalov Monte Carlo calculations.

**Table A6: Method 3A absorbed dose to water calculated from reference air kerma rate for the modified audit phantom in water**

| Quantity | Symbol | Value | Uncertainty u | Units | Type | Standard u in D_w_ (%) |
| --- | --- | --- | --- | --- | --- | --- |
| Reference air kerma rate (at 1 m) | *AKR* | 5.31 × 10^-6^ | 8 × 10^-8^ | Gy s^-1^ | B | 1.5 |
| Source-TLD distance 58.3 mm | *d* | 58.3 | 0.3 | mm | B | 1 |
| Plato^TM^ TPS calculated (Daskalov) factor* | *k_Plato_* | 317.95 | 6 | - | B | 2 |
| Exposure time per dwell position | *t* | 98.8 | <0.1 | s | B | <0.1 |
| Number of dwell positions | *n* | 6 | 0 | - | B | 0 |
| (Subtotal) Absorbed dose to water | *D_w_* | 1.000 |  | Gy |  | 2.7 |
| TLD readout (average of 9 rods) |  | 3552.52 | 24.9 | nC | A | 0.7 |
| TLD calibration coefficient |  | 2.81 × 10^-4^ |  | Gy/nC |  | 2.8 |

**Table A7: Method 3B absorbed dose to water calculated from ionisation chamber in the modified audit phantom in water**

| Quantity | Symbol | Value | Uncertainty u | Units | Type | Standard u in D_w_ (%) |
| --- | --- | --- | --- | --- | --- | --- |
| Ion chamber reading at 58.3 mm in water, cap off | *M_u_* | 1.79 × 10^-8^ | 2 × 10^-11^ | C | A | 0.1 |
| Correction for temperature, pressure, electrometer | *k_TP_.k_elec_* | 1.015 | 0.001 | - | B | 0.1 |
| Positioning of ion chamber relative to TLDs | *d* | 58.3 | 0.3 | mm | B | 1 |
| Ion chamber calibration coefficient from ARPANSA | *N_K,Ir-192_* | 4.89 × 10^7^ | 5 × 10^5^ | Gy/C | B | 1 |
| TRS-277 corrections *k_att_.k_m_* | *k_att_.k_m_* | 0.972 | 0.002 | - | B | 0.2 |
| Correction for bremstrahlung loss | 1-g | 0.997 | 0.001 | - | B | 0.1 |
| Average stopping power ratio | *s_w,air_* | 1.137 | 0.003 | - | B | 0.3 |
| Wall correction | *p_wall_* | 0.99 | 0.005 | - | B | 0.5 |
| Central electrode correciton | *p_cel_* | 0.98 | 0.005 | - | B | 0.5 |
| Correction for non-uniformity at 58.3 mm | *k_n_* | 1.035 | 0.01 | - | B | 1 |
| (Subtotal) Absorbed dose to water | *D_w_* | 0.985 |  | Gy |  | 1.9 |
| TLD readout (average of 9 rods) |  | 3552.52 | 24.9 | nC | A | 0.7 |
| TLD calibration coefficient |  | 2.77 × 10^-4^ |  | Gy/nC |  | 2.1 |


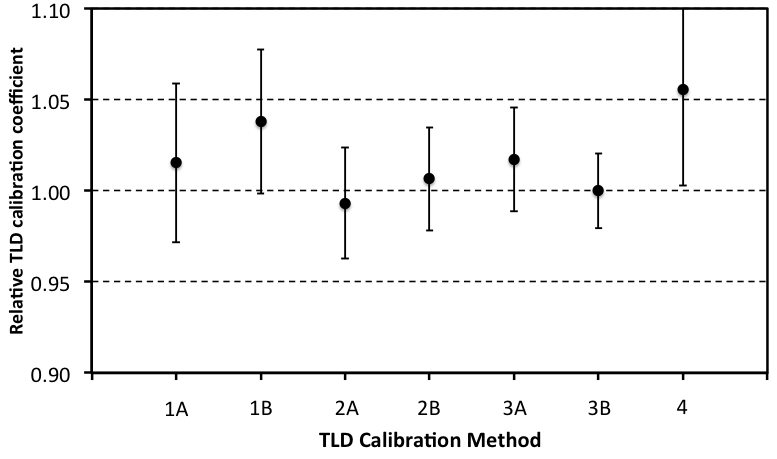


**Figure A1:** The relative TLD calibration coefficient, as determined by the different methods, normalised to the coefficient obtained using Method 3B (modified audit phantom in water).
